# Extracellular RNA as a kind of communication molecule and emerging cancer biomarker

**DOI:** 10.3389/fonc.2022.960072

**Published:** 2022-11-17

**Authors:** Danny Wu, Tao Tao, Emily A. Eshraghian, Peixu Lin, Zesong Li, Xiao Zhu

**Affiliations:** ^1^ Institute of Marine Medicine, Guangdong Medical University, Zhanjiang, China; ^2^ Department of Gastroenterology, Zibo Central Hospital, Zibo, China; ^3^ Department of Medicine, University of California (UC) San Diego Health, San Diego, CA, United States; ^4^ Guangdong Provincial Key Laboratory of Systems Biology and Synthetic Biology for Urogenital Tumors, Shenzhen Key Laboratory of Genitourinary Tumor, Department of Urology, The First Affiliated Hospital of Shenzhen University, Shenzhen Second People’s Hospital (Shenzhen Institute of Translational Medicine), Shenzhen, China; ^5^ Ningbo Institute of Life and Health Industry, Hwa Mei Hospital, University of Chinese Academy of Sciences, Ningbo, China

**Keywords:** exRNA, exosome, circulating tumor cell, cell communication, biomarker

## Abstract

Extracellular RNA (exRNA) is a special form of RNA in the body. RNA carries information about genes and metabolic regulation in the body, which can reflect the real-time status of cells. This characteristic renders it a biomarker for disease diagnosis, treatment, and prognosis. ExRNA is transported through extracellular vesicles as a signal medium to mediate communication between cells. Tumor cells can release more vesicles than normal cells, thereby promoting tumor development. Depending on its easy detection, the advantages of non-invasive molecular diagnostic technology can be realized. In this systematic review, we present the types, vectors, and biological value of exRNA. We briefly describe new methods of tumor diagnosis and treatment, as well as the difficulties faced in the progress of such research. This review highlights the groundbreaking potential of exRNA as a clinical biomarker.

## Introduction

Extracellular RNA (exRNA) is a class of nucleic acid molecules first discovered in serum and plasma (1, 2). Due to the extensive presence of RNA enzymes in the extracellular space and body fluids, experts previously deduced that exRNA could not be stable outside the cell. However, two studies (3, 4) demonstrated that RNA could be detected in microvesicles and exosomes; these structures are characterized by special membrane structures that permit the protection of the internal RNA from enzyme degradation. ExRNA can move along microvesicles and exosomes, acting as a signal molecule to affect neighboring cells or conduct long-distance regulation (5). Further, exRNA was found in almost all biological fluids, including blood, urine, and cerebrospinal fluid, and in the fluids collected in abdominal and pulmonary cavities (6–12). This is of great significance for the study of exRNA as a biomarker. A report showing a map of exRNA indicates the species carrier of exRNA in five human biological fluids: serum, plasma, cerebrospinal fluid, saliva, and urine (13). A model containing six exRNA types (CT1, CT2, CT3A, CT3B, CT3C, and CT4) indicates that each type of exRNA is associated with its carrier (vesicle and non-vesicle) (14). ExRNA transport carriers include extracellular vesicles (EVs), lipoprotein particles, and ribonucleoprotein. These exRNAs can be used as biomarkers to regulate important biological processes in the body and monitor the occurrence of various diseases (15). There is no doubt that exRNA has great potential in the field of biomarkers. If tumor biomarkers can be accurately located and detected on a non-invasive basis, exRNA may have groundbreaking impacts in the field of oncology.

EVs mainly have three forms: microvesicles, exosomes, and oncosomes. The difference between microvesicles and exosomes lies in the direction of membrane budding. Microvesicles are produced by the outward budding of the plasma membrane and released directly into the extracellular environment. In essence, exosomes are produced by the inward budding of the membrane. The first step is the inward budding of the membrane to form the multivesicular endosomes (MVEs). Then, intraluminal vesicles (ILVs) are formed in the lumen of MVEs. ESCRT components, ceramides, tetrasanins, and syntenins play a key role in this process. When the MVB membrane fuses with the plasma membrane, exosomes are released into the extracellular environment. There is also a class of specialized EVs released by tumor cells, which are called oncosomes. It is a large EV formed by cancer cells that carries several cancer-causing molecules. Studies have shown that the regulatory factors related to EV biogenesis are overexpressed in cancer, which may be related to the development, progression, and metastasis of cancer (16).

Other studies have found that the loading of extracellular RNA involves a complex sorting mechanism. RNA-binding protein (RBP) can not only bind RNA to prevent the degradation of exRNA but also play an essential role in the process of RNA targeting EVs. Some RBPs can also perform miRNA classification by recognizing specific RNA motifs. For example, hnRNPA2B1, SYNCRIP, and ANXA2 can mediate miRNA sorting and then induce the entry and localization of extracellular RNA to EVs. The regulation of miRNA sorting involves the expression level of miRNA, the sphingomyelin pathway, and the posttranscriptional modification of its 3’ terminus. The main components of miRISCs (mirNA-loaded RNA-induced silencing complex) such as Agos and GW182 can influence the sorting and loading process of miRNAs by associating with the endosomal pathway and then further mediate RNA silencing to affect the sorting and loading process of miRNA. The inhibition of neutral sphingomyelinase 2 and ceramide production can prevent multiple miRNAs from loading into EVs. In addition, uridylation and adenylation at the 3’ end of miRNAs are also associated with the release and retention of miRNAs in exosomes. When the EV-carrying information molecules meet the receptor cells, first of all, by directly triggering the membrane fusion mechanism, the contents of EVs can be directly released into the receptor cells, and finally, the transmission of information between cells can be completed. In addition, EVs can be ingested into recipient cells by corresponding endocytosis. EVs can also bind to receptors on the cell membrane, triggering intracellular signal transduction pathways, and then cascade information transmission process. When RNA is released from EVs, it can activate a series of biological functions, such as affecting protein translation (including promoting and inhibiting effects) and changing gene expression patterns (17, 18).

## Analytical methods of exRNA

### Analysis of RNA in extracellular vesicles

**Figure 1:**
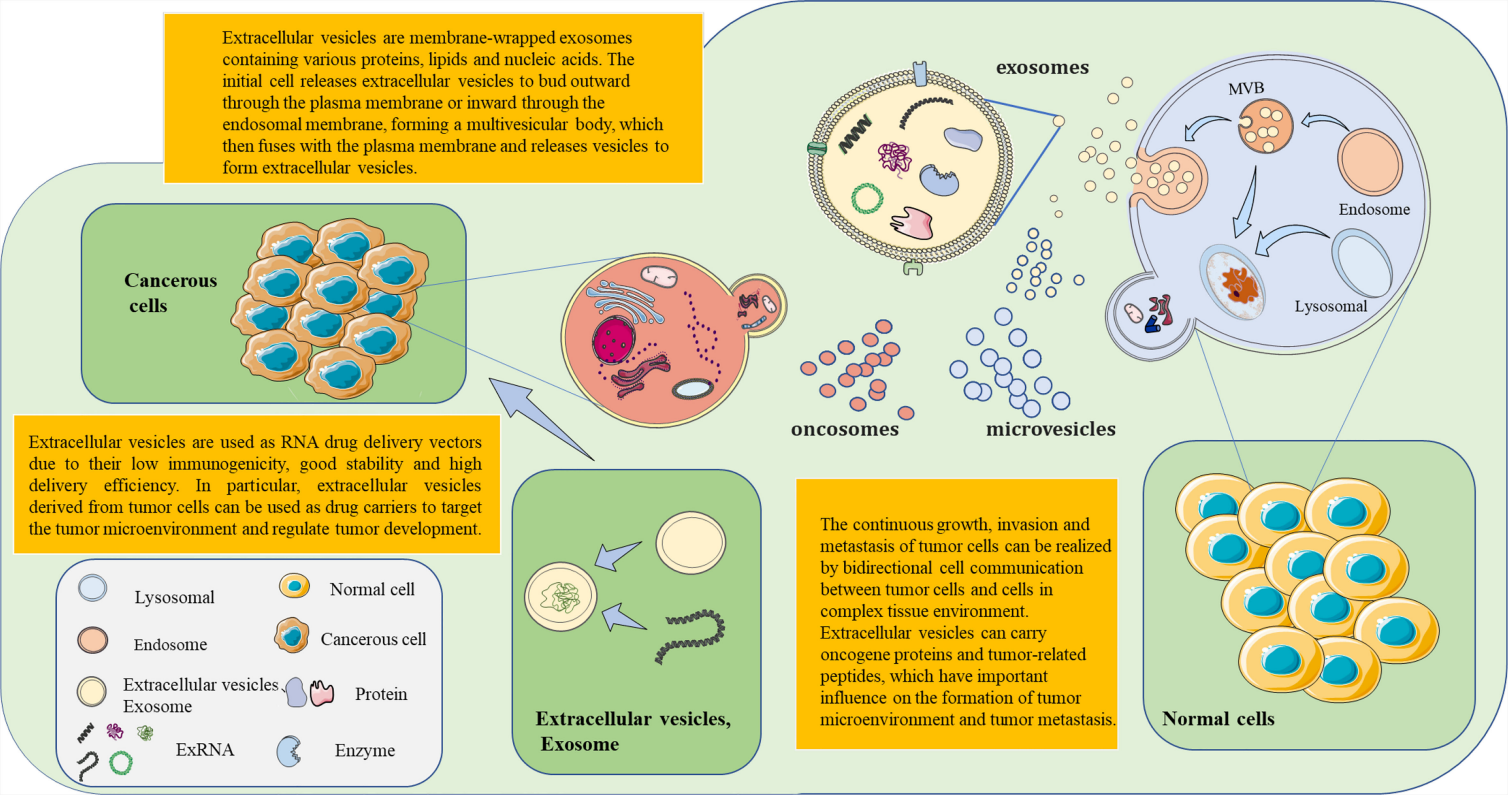
The formation of extracellular vesicles (EVs) and exosomes is based on continuous cell membrane renewal. EVs have three forms: microvesicles, exosomes, and tumor bodies. Microvesicles arise from the inward budding of the membrane. Exosomes are produced by the outward budding of the membrane. Tumor bodies are produced by tumor cells. The invagination of the cell membrane forms the endosome, which then assembles the components within the cell. This results in the formation of multivesicular bodies (MVBs). MVBs then fuse with the cell membrane. EVs within MVBs are released from the cell. EVs (exosomes) contain nucleic acids, lipids, and proteins from the source cell. These EVs, which carry information molecules, play an important role in the communication of information between cells. Based on the performance of exosomes as molecular carriers, therapeutic drugs (e.g., small interfering RNAs) are encapsulated in exosomes *in vitro* for targeted delivery to related cells. This opens a new horizon for novel precision therapy methods.

EVs can transport many types of RNA (19). These RNAs have specific biological functions and important clinical application values. In clinical disease surveillance, it is extremely important to read the RNA information in EVs. Giraldez et al. (20) developed a novel RNA sequencing method, Phospho-RNA-seq, which identifies thousands of mRNA/lncRNA fragments in plasma. This new RNA assay no longer misses critical transcriptome information and reveals the fact that the extracellular transcriptome in human biological fluids is more complex than previously known. Li et al. (21) conducted further studies on exRNA and updated the previously established exRNA database, Exorbase (www.exoRBase.org), to include more samples and data; this provides a more detailed detection and analysis for exLRs. Simultaneously, the RNA mapping program in human EVs/exosomes will be examined to 1) establish a standard protocol for the detection and analysis of exLRs and 2) construct the exLR mapping for common diseases. Uman et al. (22) proposed a natural mechanism for RNA exchange and cell-to-cell communication by using eukaryotes. EVs were used as RNA drug delivery vectors. However, the common vectors for RNA drug delivery are immunogenic or cytotoxic. The research team used human red blood cells to produce EVs for RNA therapy. Large numbers of RBCEVs deliver ASO to leukemia and breast cancer cells. The proliferation of tumor cells was reduced by the inhibition of miR-125b (22) (**Figure 1**). The further development of cancer-targeting peptides or antibody-coated RBCEVs could potentially target therapeutic RNA delivery to cancer cells and reduce side effects in normal tissues. As a potential biomarker for disease diagnosis, extracellular vesicle RNA is associated with many pathophysiological processes (23). The study of RNA in extracellular vesicles will advance new possibilities for disease surveillance and provide communities with more precise information about health and the disease status.

### Separation method of exosomes and exRNA

Exosomes are subtypes of extracellular vesicles secreted by various cell types such as stem cells, cancer cells, and immune cells. Faruqu et al. (24) successfully delivered siRNA and microRNA to target cells using exosomes. This study confirmed that exosomes can effectively deliver various biomolecules to recipient cells and play an important role in the delivery of anticancer and anti-inflammatory drugs. Due to the advantages of exosomes in drug therapy, the isolation of exosomes has become very important. The most popular method of exosome isolation is the precipitation of exosomes from the original material by hypervelocity centrifugation. With this method, protein coprecipitation usually occurs. If supercentrifugation is combined with density-based separation methods (such as the sucrose gradient), the content of impurities in isolated exosomes can be significantly reduced (25). Combining the three methods, hypercentrifugation, iodoxanol density-gradient centrifugation, and gel filtration can provide high quantities of exosomes for mass spectrometric analysis (26). However, it is important to note that the repeated centrifugation of samples may damage exosome vesicles, resulting in the poor quality of isolated exosomes. The ultrafiltration centrifugation method uses the interception of an ultrafiltration membrane with different relative molecular weights for selective separation. This method ensures the quality of exosomes, but the disadvantage is that exosome separation efficiency is low. Once exosomes block the filtration pores, the membrane life will be shortened.

In addition, adhesion between exosomes trapped on the membrane can also occur, reducing exosome yield (27). To utilize the specific markers on the surface of exosomes *in vivo* (28), researchers attempted to isolate exosomes using a magnetic bead-based immunoassay. In a clinical breast cancer biopsy study, a magnetic bead–based exosome immunoaffinity separation system was developed by using the high-affinity aptamer of CD63 protein. This system can effectively isolate exosomes from human breast cancer cell media (29). This assay fixed the captured receptor on the solid-phase carrier and targeted the exosome. Exosomes were further isolated and purified based on receptor–ligand affinity. However, this method poses challenges due to its low efficiency and potential errors considering the multistep purification processes. Based on this method, IBA Life Sciences developed an affinity traceless separation-based fragment antigen binding (FAB) method, which uses an affinity chromatography system for exosome separation without the use of magnetic beads. It works by utilizing the principle that double-stranded tags and FAB fragments (FABs) can reversibly bind and release exosomes. The application of this innovative method, with a simple and convenient purification process, has markedly improved exosome isolation as the separation reagents used in purification are reversible and have no impact on the biological activity and function of exosomes. Notably, exosomes can be isolated with high specificity and minimal contamination even in the setting of several interference factors in body fluid samples. In addition, functional materials and microfluidic chips have been used for exosome separation. This is a microfluidic exosome analysis platform based on a new GO/polydopamine (GO/PDA) nanointerface. Nanostructured GO/PDA interfaces greatly improve the efficiency of exosome immunocapture while effectively inhibiting non-specific exosome adsorption (30, 31). The innovation and development of exosome separation technology will greatly accelerate the application of exosome diagnosis and the treatment of disease (**Table 1**).

**Table 1:** Extraction and analysis of exosomes.

Technical method of separation	Characteristics	Disadvantages
Hypervelocity centrifugation [Ashley et al. *Cell* 2018]	Widely used, low cost	Challenging method with low exosome purity
Supercentrifugation combined with density-based separation [Abramowicz et al. *Mol Biosyst* 2016]	Simply method with improved exosome purity	Method is tedious and time-consuming
Hypercentrifugation combined with iodoxanol density gradient centrifugation and gel filtration [Vanaja et al. *Cell* 2016]	Can yield a large number of exosomes with high purity	Repeated complex procedures can alter exosome quality
Ultrafiltration centrifugation [Zhang et al. *J Hematol Oncol* 2015]	Exosome characteristics and quality are largely maintained	Low output rate is not conducive to a large number of analytical experiments
Separation method based on immune magnetic beads [Song et al. *Molecules* 2020]	The high specificity of immunoaffinity can isolate high-purity exosomes	Not all surface markers are clear, the reagent cost is high, and the biological activity of exosomes may be affected
Affinity traceless separation technology	The method is highly innovative and low in cost. It can obtain high-specificity and high-purity exosomes and minimize contamination	This method requires special reagents, and separation requires highly targeted conditions
Microfluidic exosome analysis platform [He et al. *Lab Chip* 2014]	The immune capture efficiency of exosomes is high, and the output of undesired exosomes is specifically inhibited	The new method requires matching reagents and instruments and has not been widely used

The exosome isolation strategies are different for different biological samples. The serum contains more exosomes than plasma because platelets release a certain amount of EVs during clotting. When studying the biology of platelets, serum samples are typically used. Plasma is recommended as a biological sample for exosome research. If exosomes in plasma are to be extracted, anticoagulants should be utilized to prevent clotting. The amount of exosomes in urine samples is lower than that in blood. Some studies have demonstrated that morning urine and midstream urine are more suitable biological samples for exosome extraction. The content of exosomes in the supernatant of the samples treated by eddy currents increased. Urine also contains specialized proteins, such as Tamm–Horsfall protein (THP). After inserting the reducing agent DTT, a large amount of EVs captured by THP is released, and the purity of exosomes can be effectively improved. In the case of breast-milk samples, the increase of dead cells after refrigeration interferes with exosome extraction. Therefore, exosomes will be extracted more efficiently by using fresher milk. Breast milk was centrifuged from high to low density, and then, the supernatant removed from fat and cells was taken to ensure further exosome extraction. If direct high-speed centrifugation is used, it is likely to cause the globulins in breast milk to agglomerate with extracellular vesicles, making the isolation of exosomes more difficult. Cerebrospinal fluid is the best biological fluid to judge the status of the central nervous system. For such small biological samples, filtration and molecular exclusion chromatography are usually more suitable.

## The role of extracellular RNA in cancer progression and its potential as a biomarker for cancer

### The role of extracellular RNA in cancer progression

Some studies have found EVs associated with tumor cells in the biological samples of cancer patients, carrying relevant carcinogenic factors and substances that can promote the occurrence and development of tumors. These factors have a profound impact on the invasion, immune escape, metastasis, and other aspects of cancer. For example, in the metastasis of breast cancer, macrophages can release EV-containing miR-223 to transmit the “invasion ability” of tumor cells from cell to cell. EV-RNA released by tumor cells can also damage the vascular endothelial barrier and cause secondary changes in endothelial permeability. Finally, it promotes the endothelial infiltration of cancer cells, leading to the enlargement of cancer cell spread, and even causes metastasis through the circulatory system. In addition, the role of EV-RNA from tumor cells in enhancing endothelial cell proliferation and angiogenesis should not be underestimated. The growth of tumor cells depends on the rich blood supply and nutrition. The pro-angiogenic effect of EV-RNA in tumor cells provides a good basis for tumor growth and metastasis to a certain extent. This essentially provides a driving force for tumor migration and invasion. In the process of cancer development, the communication of information is bidirectional, and there is mutual interference between tumor cells and normal cells. Normal cells can inhibit the proliferation of cancer cells and induce the apoptosis of cancer cells by releasing EVs rich in inhibitory miRNA, thus inhibiting the malignant phenotype of cancer cells. However, EV-RNA from certain normal cells may also help tumors grow and metastasize. For example, EV-miRNA from hypoxic mesenchymal stem cells (MSCs) can improve the survival rate of cancer cells and promote proliferation, migration, and invasion. When the gene expression of normal cells is regulated by EV-miRNA from tumor cells, a cancer-promoting effect appropriate to the biological function of cancer cells may occur, such as cancer-associated fibroblasts (CAFs) and mesenchymal stem cells in the tumor microenvironment. Once the balance between normal cells and cancer cells in the body is disrupted, cancer is endowed with the ability to metastasize and invade. The EV-RNA of cancer cells mediated the antitumor immune response by activating the function of immune cells, but it may also play an inhibitory effect to mediate the immune escape of the tumor. Under the interference of tumor EV-RNA, tumor-associated macrophages in the tumor microenvironment will change the cell phenotype and promote cancer progression. Studies have also shown that immune cells associated with hypoxia release EV-RNA, which can promote tumor growth and migration. In addition, the EV-RNA-mediated gene reprogramming of immune cells is one of the main reasons why the function of T cells is hindered, which promote the escape of cancer cells (16, 18).

### Circulating tumor markers in liquid biopsy

Currently, the main clinical screening and diagnosis methods are endoscopies, imaging detection, and tumor marker detection (32). However, these methods are restricted to specific cases and are not sensitive enough. In the treatment of tumors, an accurate and early diagnosis often results in a more hopeful prognosis; this indicates the importance of primary diagnoses of tumors. Liquid biopsy is highly sensitive to trace tumor-derived nucleic acids and other markers, and miRNA, lncRNA, and mRNA can all be used as tumor-specific markers in the clinical application (33–35). This provides a new possibility for the study of the early or even ultra-early screening of tumors. In the identification of tumor-specific gene expression profiles, miRNA is often used as the preferred biomarker for detection. The miRNAs exist in blood or are encapsulated in exosomes and are not easily degraded. Specific serum miRNA expression levels can be used for the initial screening, diagnosis, and prognostic trackings of common tumors, such as lung cancer, breast cancer, and liver cancer. The scope of action of lncRNA includes almost all physiological and pathological processes, and it plays an important role in regulating gene silencing and gene expression. LncRNA has high stability in blood, and its high abundance compared with circulating tumor cells and cell-free DNA shows its potential as a more reliable tumor marker. In the current study, some lncRNAs that are closely related to the tumor diagnosis and prognosis have been detected in lung cancer, liver cancer, gastric cancer, and prostate cancer. Circulating lncRNA levels can reflect the existence of tumors in the body to a certain extent. This is of great significance for the early screening and diagnosis of tumors in the future.

## exRNA in tumor growth, metastasis, and metabolism

Studies have shown that host cells or tumor cells are involved in tumor genesis, growth, invasion, and metastasis through special signal transduction modes (36, 37). During cell growth and development, the cell envelope invaginates to form multiple vesicles. After a series of physiological effects in the cell, it finally fuses with the cell membrane and then releases, forming exosomes containing a signal medium (38–42). EVs can mediate intercellular communication in different ways after secretion (**Figure 1**). Protein molecules or lipid ligands on the surface of exosomes directly activate receptors on the surface of target cells, generating signaling complexes and activating intracellular signaling pathways. The researchers engineered the surface of the cell membrane to drive the antiviral signal in the receptor breast cancer cell in a protein-free binding form, resulting in tumor growth (43, 44).

In addition, exosomes can fuse with target cells or enter the target cells through endocytosis, bringing their proteins, nucleic acids, ​lipids, and other active molecules into cells to regulate the function and biological ​behavior of cells. Lunavat et al. (45) determined the amount of EV RNAs released by cancer cells after treatment with verofenib. The increased expression of miR-211-5p was ​observed in EV secreted by verofenib-treated cells and tumor tissues ​from xenografted tumor patients. In conclusion, the treatment of verofenib can induce the upregulation of miR-211-5p in melanoma cells *in vitro* and *in vivo*. Tumor-derived exosomes contain components associated with tumor cell miRNAs and have demonstrated the ability to independently process precursor miRNAs into mature miRNAs (46). Roccaro et al. (47) collected many oncogenic proteins, cytokines, and kinases in exosomes isolated from bone marrow stromal cells in myeloma.

These tumor-associated miRNAs and proteins are transported by exosomes, creating an environment that favors tumor metastasis. Interestingly, certain properties of exosomes prompt novel possibilities in the treatment of cancer. Pi et al. (48) found that attaching antibody-like (i.e., Y-shaped) RNA nanoparticles to microvesicles can deliver effective RNA therapeutic agents such as small interfering RNA (siRNA) specifically to cancer cells. By using RNA nanotechnology, researchers have successfully generated extracellular vesicles capable of successfully targeting three types of cancer therapeutics in animal models. Further, its influence on the metabolism of tumor cells cannot be underestimated. Chen et al. (49) investigated the mechanism of the aerobic glycolysis of breast cancer tumor cells with a high quantity of macrophages. This study demonstrates that tumor-associated macrophages can transmit signals through EVs containing lncRNA. This signal enhances the aerobic glycolysis of breast cancer cells and improves the ability of antiapoptosis. The study also highlights the potential of lncRNAs as signal transducers, which propagate between immune cells and tumor cells by EVs and promote the aerobic glycolysis of tumor cells.

In summary, the growth, metastasis, and metabolism of tumors have a significant impact on the subsequent treatment of tumors. Fundamentally understanding the biological characteristics of tumor cells and further research on tumor biomarkers can significantly impact the treatment of tumors, bringing a renewed sense of hope to patients with tumors.

## Applications in cancer research

### Lung cancer

Cigarettes and environmental pollution are two major culprits of the high incidence of lung cancer. Lung cancer accounts for a high proportion of cancers, among which lung squamous cell carcinoma (LUSC) and lung adenocarcinoma (LUAD) are the main subtypes (50–52). Cheng et al. (53) performed a circRNA detection of ​LUSC and its adjacent normal tissues by using the ceRNA chip. Through coexpression analysis and qPCR verification, the target circRNA-circTp63 and the target gene FOXM1 of circTp63 were first identified. In further bioassay experiments, circTp63 was considered to promote the cell cycle progression by acting on FOXM1 and CENPA and CENPB downstream genes through miR-873-3p. It ultimately promotes the proliferation and tumor growth of LSC cells. Nigita et al. (54) combined miRNA ​sequencing data from 87 samples of non-small cell lung cancer (NSCLC) and 26 independent exosomes derived ​from plasma to investigate RNA editing. This experimental data suggest that there is an editing event in the dysregulated microRNA between the tumor and normal tissue in NSCLC. The fifth edit of miR-411-5p was significantly dysregulated in the tissues and plasma exosomes of patients with NSCLC. Hayashita et al. (55) found that miR17-92 was also associated with lung cancer, and this miRNA was overexpressed in most lung cancer cell lines. When exogenous MIR17-92 was introduced, the growth of lung cancer cells was significantly promoted. Liu et al. (56) performed differential lncRNA screening on spinal samples from patients with primary lung adenocarcinoma, patients with spinal metastases (SM) from lung adenocarcinoma, and normal controls. Linc00852 and MAPK pathways were associated with lung adenocarcinoma SM. In addition, *in vivo*, and *in vitro* experiments have shown that the target gene S100A9 of LINC00852 has a positive regulatory effect on the growth, migration, invasion, and metastasis of lung adenocarcinoma cells. S100A9 strongly activates P38 and Rek1/2 kinases and slightly activates JNK kinase phosphorylation in the MAPK pathway in A549 (human alveolar basal epithelial cells) and SPCA-1 cells, thereby promoting the progression and oncogenic ability of SM in lung adenocarcinoma. This suggests that SM intervention is a potential novel therapeutic target in the early stage of lung cancer. Further, evidence suggests that exRNA has a strong potential as a diagnostic marker for lung cancer. In future research on lung cancer, the detection of exRNA expression profiles in lung cancer tissue is expected to become the principal force of non-invasive detection methods (**Table 2**).

**Table 2:** Functional effects of extracellular RNA (exRNA) shuttled by extracellular vesicles.

Cancer	ExRNA	Downstream targets	Effect or significance
Lung cancer	circTP63	FOXM1	Promotes cell proliferation and tumor growth *in vitro* and *in vivo*
	miR-411-5p		Potential metastatic targets related to lung cancer biology
	miR17-92		Enhanced lung cancer cell growth
	LINC00852	S100A9/MAPK	Promote the progression and oncogenic ability of lung adenocarcinoma SM
ESCC	GLM1-NAA35 RNA		Potential biomarkers of esophageal cancer (saliva)
GC	miR-551b-3p	TMPRSS4	Inhibit the proliferation, migration and invasiveness of gastric cancer cells
	LncRNA SMARCC2	miR-551b-3p	Enhanced the proliferation and migration of gastric cancer cells
	circ-RanGAP1	miR-877-3p/VEGFA	Enhanced the migration and invasion potential of GC cells
	miR-196、miR-92、miR-1307		Promote the dissemination of peritoneal tumor cells
HCC	has_circ_0025129	miR-34a、USP7/Cyclin A2	Reduces the DNA damage and promotes tumor growth
	CircPTGR1	miR449a–MET	Increase the migration and invasion ability of tumor cells
	miR-21	PTEN、PTENp1、TETs	Promote hepatocellular carcinoma growth
	miR-23a-3p	PTEN-AKT、PD-L1	Promote the escape of tumor cells from immune surveillance
PC	miR-21-5p、miR-200c-3p		Relatively better differentiating between prostate cancer and prostatic hyperplasia
	Let-7a-5p		To distinguish prostate cancer patients with Gleason score ≥8 vs. ≤6
Breast cancer	miR-106a		Potential biomarker for metastatic breast cancer
	hsa_circ_001783	miR-200c-3p	Enhanced the proliferation and metastasis of breast cancer cells
Ovarian cancer and endometrial cancer	miR21	APAF1	Endowing ovarian cancer cells with paclitaxel resistance
	hsa_circ_0109046、hsa_circ_0002577		It may assist in predicting the occurrence and metastasis of endometrial cancer
GBM	circNT5E	miR-422a、Akt、Smad2	Promote glioblastoma tumorigenesis
MM	LINC00461	miR-15a/16、Bcl-2	Promoted multiple myeloma cell proliferation and inhibited cell apoptosis

### Esophageal squamous cell carcinoma

Esophageal squamous cell carcinoma (ESCC) is the sixth leading cause of cancer death worldwide (57, 58). To advance the study of non-invasive biomarker detection, Lin et al. (59) extracted and analyzed the G-Nchirna in exosomes from the tissues and saliva of mice and ESCC patients. The clinical potential of salivary exosomes G-Nchirna (SEG-Nchirna) as readily accessible biomarkers was assessed. The G-Nchirna of exosomes can be detected in ESCC cells and xenograft tumor models in nude mice. The tumor load was closely related to SEG-Nchirna levels. This study reveals that SEG-Nchirna can be used as a reliable biomarker to evaluate the initial detection, treatment response, and recurrence of ESGG (**Table 2**).

### Gastric cancer

Gastric cancer (GC) is a malignant disease with high morbidity and fatality rates (60). Yuan et al. (61) measured the average expression of miR-551b-3p in patients with GC utilizing a quantitative reverse-transcription polymerase chain reaction experiment. In the study samples, the expression of miR-551b-3p was low in 64% of cases. Further analysis indicates that patients with low miR-551b-3p expression versus patients with high miR-551b-3p expression demonstrate varying prognoses. In addition, it was found that LncRNA SMARCC2 can act as an upstream regulator of Mir-551b-3p, thereby enhancing the proliferation and migration of GC cells by inhibiting the expression of MiR-551b-3p. Lu et al. (62) found that circ-Rangap1 was significantly upregulated in both the plasma exosomes and GC tissues of patients with GC. The high expression of circ-Rangap1 is closely related to TNM staging. The level of circ-Rangap1 in the plasma exosomes of patients with GC before surgery is upregulated, which enhances the migration and invasion ability of GC cells. Circ-Rangap1 may be a potential prognostic biomarker for GC and a therapeutic target for GC. Peritoneal dissemination was the most frequent metastatic method for GC. Hu et al. (63) found higher expression levels of miR-196, miR-92, and miR-1307 in the ascites-derived exosomes of GC patients. Moreover, the invasion of GC cells was closely related to the exosomes detected in the experiment. Taken together, these results demonstrate the significance of exRNA in the diagnosis, treatment, and prognosis of GC (**Table 2**).

### Hepatocellular carcinoma

**Figure 2:**
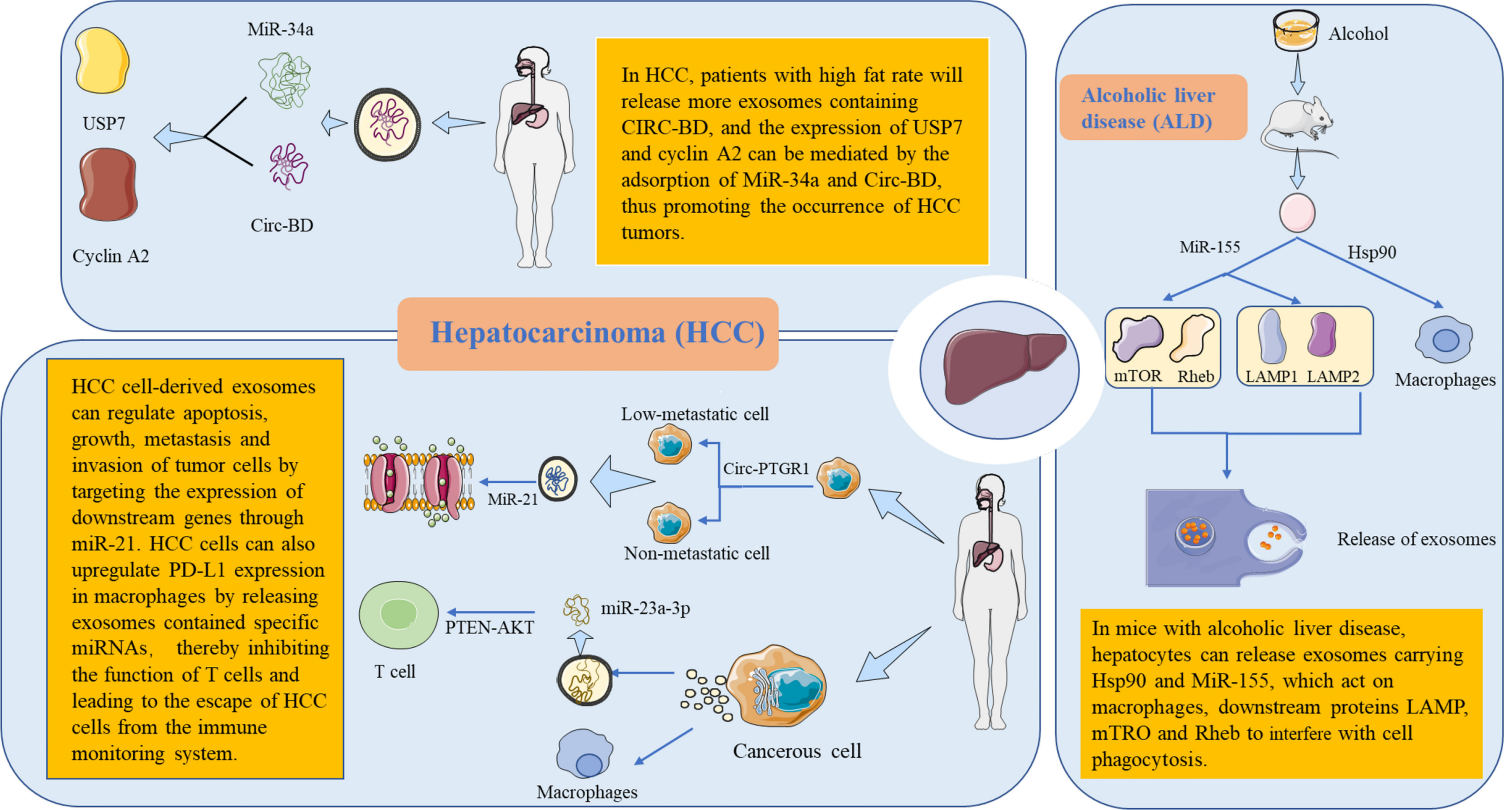
Metabolic functions and mechanisms of extracellular RNA (exRNA) in hepatocellular-derived exosomes. The liver is the body’s largest detoxification organ. In mice with alcoholic liver disease, liver cells interfere with the cellular function by releasing exRNA-carrying exosomes. HSP90 targets to macrophages and affects phagocytosis. MiR-155 acts on downstream proteins LAMP, MTRO, and RHEB, affecting autophagy through specific activation pathways and then interfering with the release of exosomes. In patients with hepatocellular carcinoma (HCC), it was found that patients with a high fat percentage released more exosomes containing circ-BD. Circ-BD was highly expressed in adipocyte exosomes. It promotes HCC tumorigenesis by absorbing miR-34a to mediate the expression of USP7 and cyclin A2. In the process of tumor cell invasion, non-metastatic and low-metastatic cells can obtain the ability of migration and invasion through exosomes containing circ-ptgr1. In addition, HCC cell–derived exosomes can increase the expression of miR-21, which regulates the apoptosis, growth, metastasis, and invasion of tumor cells by targeting the expression of downstream genes. Endoplasmic reticulum–stressed HCC cells raise the expression of PD-L1 in macrophages by releasing exosomes containing specific miRNAs. Subsequently, the miR-23a-PTEN-Akt pathway inhibits the function of T cells, leading to the escape of HCC cells from the immune monitoring system. PD-L1, programmed death-ligand 1.

The high morbidity and mortality of hepatocellular carcinoma (HCC) grants urgency to the development of more effective treatment programs for the disease (64–66). Zhang et al. (67) used ceRNA microchips to detect the expression of circRNA in the exosomes of HCC patients with high and low body fat percentages (BFRs). The results showed that the high expression of circ-BD in patients with high BFR played a certain role in promoting the tumor development of HCC. Wang et al. (68) studied the potential role of the circRNA of HCC exosomes in tumor cell migration and invasion, helping to clarify its potential mechanism. Signal transmission between cells with low or no metastatic potential and HCC cells with high metastatic potential can be achieved through exosomes. In another study, Cao et al. (69) selected HCC cells with non-metastatic (HepG2), low metastatic (97L), and highly metastatic (LM3) potential and sequenced the circRNAs in the exosomes. HCC cell–derived exosomes affected the expression of miR-21. By interfering with the expression process of downstream genes, it can regulate the physiological function of tumor cells (69). Liu et al. (70) found that endoplasmic reticulum–stressed HCC cells acted on macrophages by releasing exosomes containing specific miRNAs. It can also inhibit the function of T cells by affecting cell signal transduction pathways, leading to the escape of HCC cells from the immune monitoring system (**Figure 2**). From the direction of immunology, strengthening the immune monitoring function in the tumor microenvironment may establish new methods for the treatment of HCC (**Table 2**).

### Prostate cancer

In 2008, Kroh et al. first demonstrated the presence of miRNAs released from prostate cancer (PC) cells in the blood (71). The greatest clinical need in PC surveillance is to find reliable, non-invasive tools to distinguish between PC and benign prostate disease. Mitchell et al. (72) developed a “miRScore” based on serum levels of 14 miRNAs. Local and metastatic resistant prostate cancer (mCRPC), or low-grade and high-grade PCs, could be distinguished by analysis of free cell-free miRNAs. The results showed that some free acellular miRNAs were predictive in the differentiation between PC and benign prostatic hyperplasia (BPH). However, only a few miRNA biomarkers have been validated in multiple independent studies. Many other miRNAs have no definitive results and have shown conflicting results in some studies. The analysis of miRNA is thus regarded as a non-repeatable method. As research continues, cancer-derived EVs contain highly specific protocellular biomarkers and protect their RNA from RNA enzyme degradation in blood. Therefore, EV-based miRNA analysis may be superior to whole plasma/serum analysis. Endzelins et al. (73) determined the RNA isolated from plasma EV samples from 77 patients. The results showed that four miRNAs showed potential for the diagnosis of PC, and there were significant differences between the miRNAs encapsulated in EVs and the miRNA profiles in whole plasma. Only a small fraction of plasma acellular miRNAs was present in plasma EVs. MiR-375 was able to distinguish PC versus BPH in the whole plasma assay, while miR-200c-3p and miR21-5p were better in plasma EVs. The level of let-7a-5p in non-plasma EVs could distinguish PC patients with a Gleason score ≥8 vs. ≤6 (40), but plasma let-7a-5p had no diagnostic value. The prostate-specific antigen (PSA) test is currently recognized as the best method for early suspected prostate cancer. However, elevated PSA levels are not unique to prostate cancer and can also be caused by benign prostate disease. Therefore, in clinical practice, histopathology is generally obtained through the systemic needle biopsy of the prostate to make the definitive diagnosis (74–76). However, invasive procedures pose several challenges for patients. Non-invasive biomarker research is a pioneering field for the diagnosis and treatment of prostate cancer (**Table 2**).

### Breast cancer

Breast cancer in women surpassed lung cancer for the first time as the most common cancer globally in 2020, accounting for approximately 11.7% of incident cancer cases, according to the latest data on the global burden of cancer for 2020 released by the International Agency for Research on Cancer of the World Health Organization (77). Circulating miRNAs are formed by the nuclease degradation of RNA, which are then assembled by microvesicles and exosomes for transport. Zhao et al. (78) amplified miRNA by reverse-transcription PCR (RT-PCR) and analyzed miR-106a in blood by combining it with the miRNA microarray. It was found that the abnormal expression of miR-106a could be a potential biomarker for metastatic breast cancer. Liu et al. (79) designed a set of systematic screening methods for breast cancer–specific circRNAs and found that hsa_circ_001783 regulates tumor proliferation and metastasis through sponge miR-200C-3p. This study indicates that hsa_circ_001783 may be a novel prognostic marker and therapeutic target for breast cancer. The treatment of breast cancer has developed from simple surgical treatment to a combination of surgery, chemotherapy, radiotherapy, hormonal therapy, and/or targeted therapy. This makes breast cancer treatment more personalized and continuous. It is suspected that researching breast cancer at the molecular level will bring advantages for the screening, treatment, and prognosis of breast cancer, but this remains to be explored (**Table 2**).

### Ovarian cancer and endometrial cancer

Advanced ovarian cancer usually spreads to the visceral adipose tissue of the greater omentum (80, 81). Au et al. (82) identified and compared microRNA-21 from exosomes in ovarian cancer cells, cancer-associated adipocytes (CAAs), and cancer-associated fibroblasts (CAFs). Our results suggest that miR21 can metastasize from CAAs or CAFs to cancer cells and then inhibit ovarian cancer cell apoptosis and enable cancer cells to acquire drug resistance by binding to APAF1. In the retinal tumor microenvironment, exosomes secreted by adjacent stromal cells alter the malignant phenotype of metastatic ovarian cancer cells by the delivery of miR21. The inhibition of miR21 metastasis from a stroma has a promising application prospect in the treatment of metastatic and recurrent ovarian cancer. Endometrial cancer most often occurs in the endometrial epithelium of menopausal and postmenopausal women. Xu et al. (83) isolated circRNA in EVs from the serum samples of three patients with stage III endometrial cancer aged 50–60 years and three healthy subjects. A total of 209 circRNAs were upregulated, and 66 circRNAs were downregulated. The abnormal expression of two circRNAs, hsa_circ_0109046 and hsa_circ_0002577, was confirmed by RT-qPCR. These findings help predict the occurrence, metastasis, and prognosis of endometrial cancer (**Table 2**).

### Human glioblastoma

The intercellular communication between tumor and host microenvironment can be mediated by exosomes. Wei et al. (84) studied the cancer-derived exRNA by using glioblastoma (GBM) as a research model. The study provides a variety of categories of exRNA that can be used for biomarker discovery. At the same time, the miRNA with the greatest influence on GBM was also predicted. It provides additional possibilities for the study of biomarkers in cerebrospinal fluid. Wang et al. (85) screened circRNAs differentially expressed in GBM clinical samples by using the ceRNA microarray. The target circNT5E was identified by TargetScan prediction and an miRNA pulldown experiment. The phenotypic analysis *in vivo* and *in vitro* showed that AdARB2 can act on linear NT5E precursors and relieve ADAR-1’s inhibition of circnt5E transcription. The highly expressed circnt5E can be adsorbed to miRNA-422a to relieve its inhibition of downstream target genes. Then Akt, Smad2, and other signaling pathways are activated to promote the proliferation, migration, and invasion of GBM cells. In general, exRNA can regulate the occurrence and development of GBM through regulatory intervention on downstream target genes. This is a novel mechanism for the study of GBM (**Table 2**).

### Multiple myeloma

Multiple myeloma (MM) is a hematologic cancer caused by the abnormal expansion of plasma cells. MM is characterized by the malignant proliferation of monoclonal plasma cells and the secretion of large amounts of monoclonal immunoglobulins, which then inhibit the normal function of both polyclonal plasma cells and polyclonal immunoglobulins (86–88). Deng et al. (89) found that mesenchymal stem cell–derived exosomes promoted MM tumorigenesis by regulating the function of miR-15a/16 and Bcl-2 through lncRNA LINC00461. The expression profile analysis of lncRNA in bone marrow plasma cells showed that the dysregulation of lncRNA UCA1 was associated with the abnormal serum levels of albumin and M protein. In addition, lncRNA metastases–related MALAT1 overexpression was found in newly diagnosed MM patients compared with healthy individuals after treatment. This suggests that MALAT1 could be used as a marker to predict disease progression. The overexpression of specific lncRNA MSL1 was detected in 40% of MM samples. The knockdown of MSL1 by short hairpin RNAs significantly increased apoptosis in MM cell lines, suggesting that MSL1 may be a relevant therapeutic target. In conclusion, these data suggest that the changes in lncRNA expression are associated with the occurrence of MM (**Table 2**).

## Conclusions, challenges, and perspectives

This review introduces the great potential of exRNA as a novel biomarker. Each study of the formation process of exRNA and the complex mechanism of its role in body diseases provides new possibilities for the detection and treatment of future diseases. At present, most clinical diagnoses of the disease still include invasive procedures, such as biopsies. Based on the special physiological characteristics of exRNA, researchers foresee much potential in utilizing exRNA for non-invasive diagnostic methods (5, 8). In addition, as studies continue to deepen, there is evidence that exRNA has a greater functional value. The use of extracellular vesicles to transport RNA drugs to treat diseases is increasingly widely studied (90). Whether in the detection, diagnosis, treatment, or prognosis of diseases, exRNA has highlighted a great advantage.

The study of exRNA could lead to the search for new diagnostic markers or new therapeutic targets. However, before translating its use into clinical practice, the mechanisms and pathways of exRNA treatment should be understood. It is still unknown how to introduce this biomarker into clinical disease surveillance (10, 91, 92). Further, interspecies exRNA transfer may occur through contact and feeding, opening a new entry point for the transboundary transfer of exRNA. Although information exchange and transmission serve as a bridge between species, it is not fully understood whether or how they play an important role in biological interactions (90, 93, 94).

At present, there has been the formation of an exRNA database (95–98). The existence of a wide spectrum of exRNA and its diversity suggests that it may be involved in important biological processes, such as the regulation of normal growth and development, as well as the occurrence of cancer and disease. The study of the mechanisms behind the regulatory functions of exRNA will provide new insights into the development of certain diseases (99, 100). However, the maturity of the new technology needs to go through a perfect development process, and the extensive scope of exRNA has brought some difficulties to its in-depth research. Exosome extraction and purification have a great impact on the study of exRNA to a large extent (101, 102). Therefore, from the study of exRNA to its practical application, it still needs to be verified in practice. It is believed that with the development of science and extensive research efforts, exRNA will bring widely impact science and medicine as a whole. The prospect of the clinical application of exRNA should be more anticipated.

## Author contributions

XZ and ZL conceived the work. DW wrote and drafted the manuscript. TT, PL, ZL, and XZ discussed the manuscript. EE revised the manuscript. All authors read and approved the final version of the manuscript.

## Funding

This work was supported partly by The National Natural Science Foundation of China (81972366); The Natural Science Foundation of Guangdong (2022A1515012606); The Shenzhen Science and Technology Innovation Commission projects (JCYJ20220818101808018, KCXFZ202110203002406).

## Conflict of interest

The authors declare that the research was conducted in the absence of any commercial or financial relationships that could be construed as a potential conflict of interest.

## Publisher’s note

All claims expressed in this article are solely those of the authors and do not necessarily represent those of their affiliated organizations, or those of the publisher, the editors and the reviewers. Any product that may be evaluated in this article, or claim that may be made by its manufacturer, is not guaranteed or endorsed by the publisher.

## Glossary

**Table:**

ASO	antisense oligonucleotide
BFR	body fat rate
BPH	benign prostatic hyperplasia
CAAs	cancer-associated adipocytes
CAFs	cancer-associated fibroblasts
ESCC	esophageal squamous cell carcinoma
EV	extracellular vesicles
exLR	extracellular vesicle long RNA
exLRs	extracellular vesicle long RNAs
exRNA	extracellular RNA
Fab	fragment antigen binding
GBM	glioblastoma
GC	gastric cancer
HCC	hepatocellular carcinoma
LUAD	lung adenocarcinoma
LUSC	lung squamous cell carcinoma
mCRPC	metastatic castration-resistant prostate cancer
MM	multiple myeloma
PC	prostate cancer
PSA	prostate-specific antigen
PSC	primary sclerosing cholangitis
qRT-PCR	quantitative reverse-transcription polymerase chain reaction
RBP	RNA-binding protein
RBCEVs	red blood cell extracellular vesicles
RT-PCR	reverse transcription-PCR
RT-qPCR	real-time quantitative reverse-transcription PCR
SEG-NchiRNA	salivary exosome G-NchiRNA
SM	spinal metastases
